# Surgery for diaphragma sellae meningioma: how I do it

**DOI:** 10.1007/s00701-020-04581-6

**Published:** 2020-09-18

**Authors:** Amani Belouaer, Daniele Starnoni, Roy Thomas Daniel

**Affiliations:** 1 Department of Neurosurgery, University Hospital of Lausanne (CHUV), rue du Bugnon 46, 1011 Lausanne, Switzerland; 2 University of Lausanne, Lausanne, Switzerland

**Keywords:** Diaphragma sellae meningioma, Transcranial approach

## Abstract

**Background:**

Surgery for diaphragma sellae meningiomas (DSM) remains challenging due to the intimate neurovascular relationships of the tumor. Excision of DSM along with a decompression of the optic apparatus requires a good knowledge of the skull base anatomy and a precise preoperative evaluation of the tumor extensions.

**Method:**

We describe the key steps of transcranial approach for DSM with a video illustration. The surgical anatomy is described along with the advantages and limitations of this approach.

**Conclusions:**

The transcranial approach allows a safe tumor excision with an early and adequate control of the neurovascular structures, while minimizing postoperative CSF rhinorrhea.

**Electronic supplementary material:**

The online version of this article (10.1007/s00701-020-04581-6) contains supplementary material, which is available to authorized users.

## Relevant surgical anatomy

**Fig. 1:**
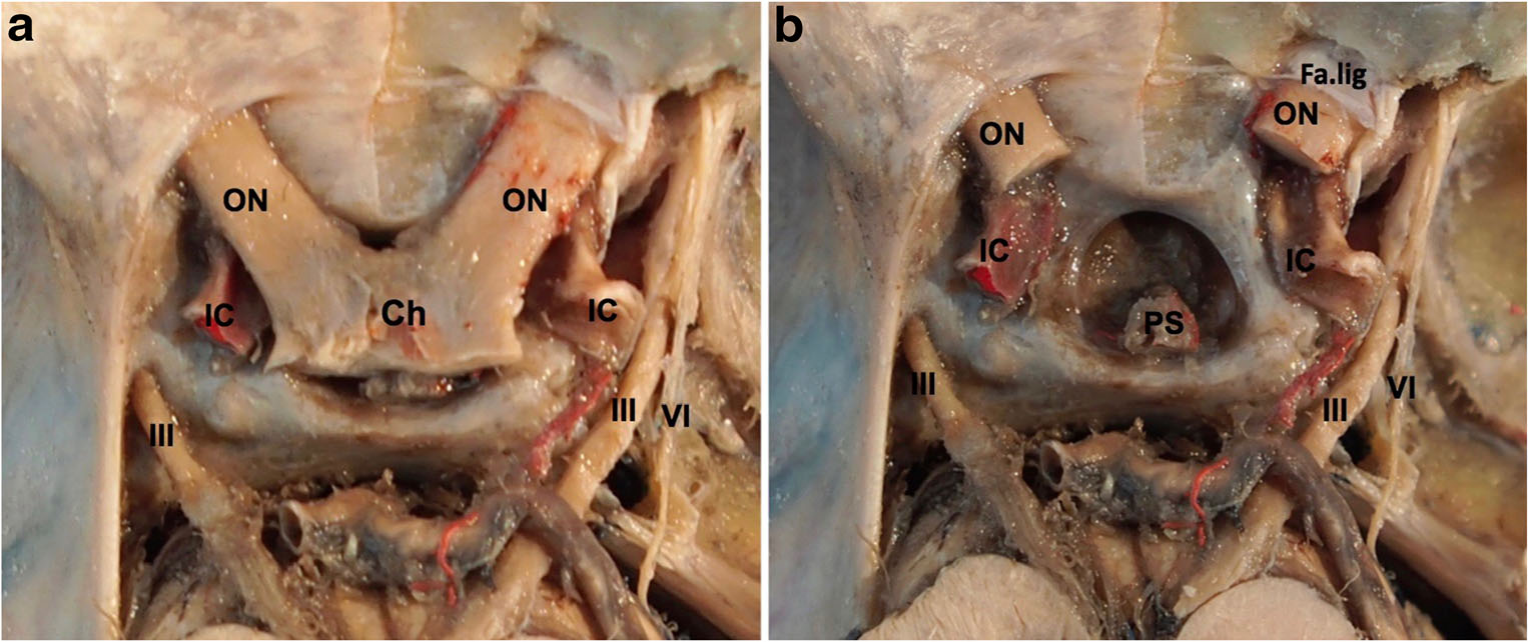
Cadaveric dissections showing the disposition of neural and vascular structures around the diaphragma sellae. Chiasm lies on the diaphragama sellae (**a**). After the removal of the chiasm, **b** diaphragma sellae and pituitary stalk can be identified. IC internal carotid artery, ON optic nerve, III oculomotor nerve, IV trochlear nerve, Ch chiasm, Fa.lig falciform ligament

**Fig. 2:**
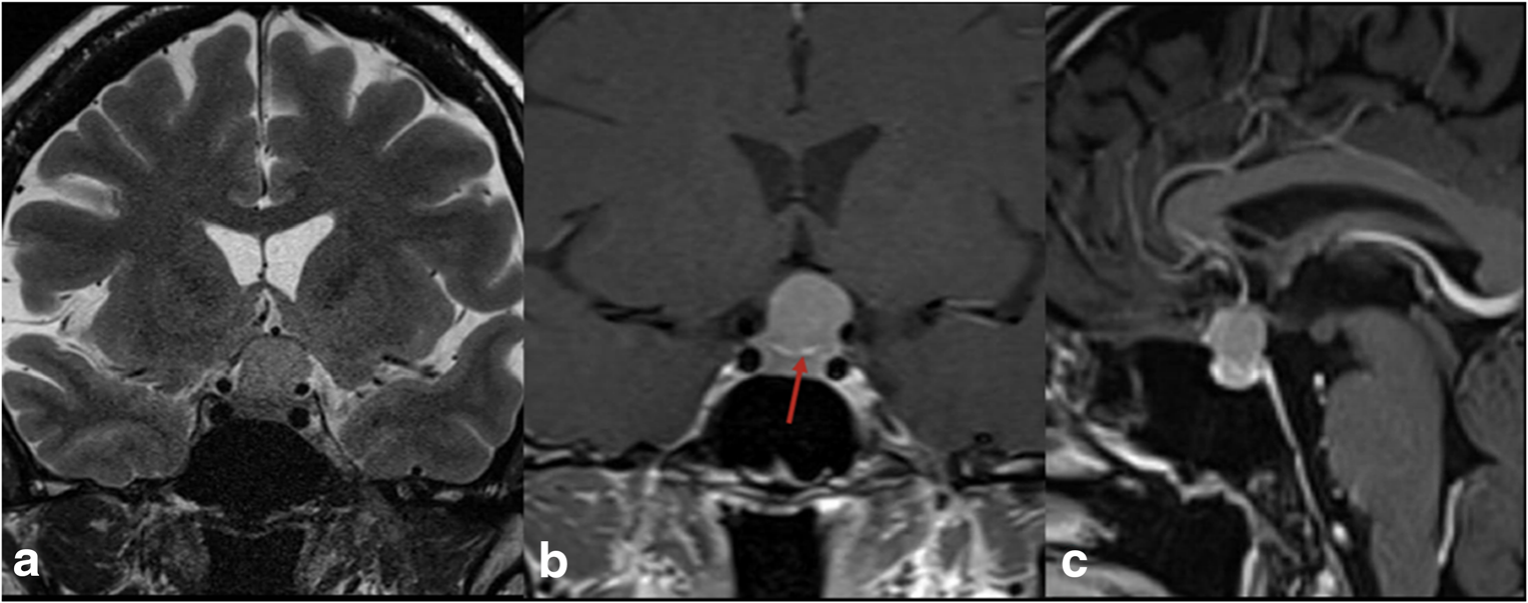
Preoperative MRI of a 45-year-old lady who presents with progressive decrease in visual acuity of the left eye. **a** T2W coronal MR image showing a homogenous tumor involving the suprasellar region extending laterally to the left internal carotid and superiorly to the chiasm which is compressed by the lesion. **b** Gd-enhanced T1 W coronal MR image showing the suprasellar lesion with enhancement of the diaphragma sellae (red arrow) separating the lesion from sellar contents. **c** Gd enhanced T1W sagittal MR image showing the widest attachment base of the tumor localized at the diaphragma sellae

The diaphragma sellae (DS) forms the roof of the sella turcica and is continuous with the dura mater covering the tuberculum sellae, the dorsum sellae, and laterally the roof of the cavernous sinus (Figs. 1b and 2) [1]. The pituitary stalk (PS) passes through a defect within the DS (Fig. 1b) [1]. The two optic nerves (ON) converge onto the optic chiasm, overlying the DS. At the level of the optic canal (OC), the ON is covered by the falciform ligament, which extends from the anterior clinoid process (ACP) to the planum sphenoidale (Fig. 1). The roof of the OC is formed by the medial root of the ACP, and the inferolateral wall is formed by the optic strut, which separates the OC from the superior orbital fissure (SOF). The internal carotid artery (ICA) pierces the dura at the distal dural ring inferomedial to the ACP and courses in a posterolateral direction. There are often (70%) two groups of superior hypophyseal arteries (SHAs), arising from ICA, which run in the preinfundibular and retroinfundibular spaces [10].

## Description of the surgical technique (video)


ESM 1(MP4 222,793 kb)

The patient is placed in the supine position, and the head turned 20°–30° to the opposite side, extended to allow easier frontal lobe retraction. A frontotemporal skin incision is carried down sharply to the pericranium. A subfascial dissection is performed, and the temporalis muscle is retracted inferolaterally. Fat is harvested from the infratemporal fossa for skull base repair and optic neuropexy, if needed. A frontotemporal craniotomy is performed with the anterior cut flush to the anterior skull base. To achieve an adequate ON decompression and prevent extensive manipulation of the nerve during tumor removal, an extradural clinoidectomy and opening of the OC is performed.

Key steps for extradural clinoidectomy [3, 9]:

- The greater and lesser sphenoid wings are resected under continuous irrigation
- The SOF is exposed and unroofed.
- The meningoorbital band is sectioned to allow an inter-dural mini peeling of its upper fold to expose the entire surface of the ACP [4].
- The diamond burr is used to core out the central cancellous bone and “egg-shelling” of the remaining cortical bone of the ACP.
- A 180° optic foraminotomy is performed.

**Fig. 3:**
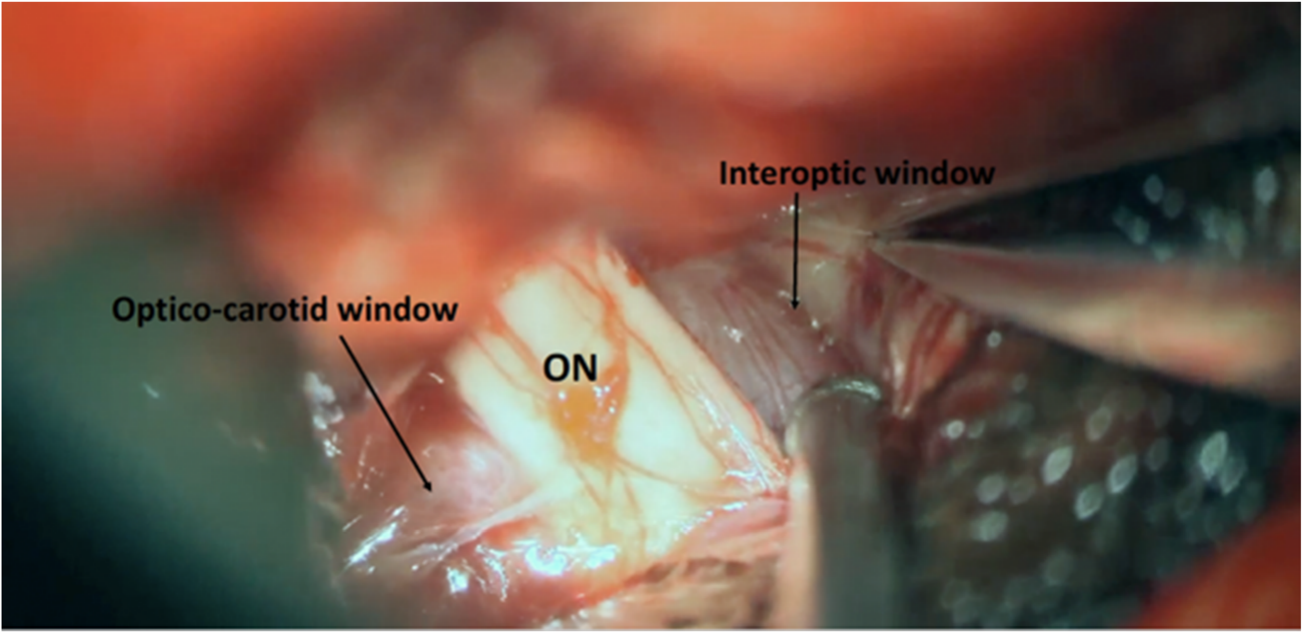
Intraoperative image showing the meningioma and the two-working windows used for tumor excision minimizing ON mobilization

**Fig. 4:**
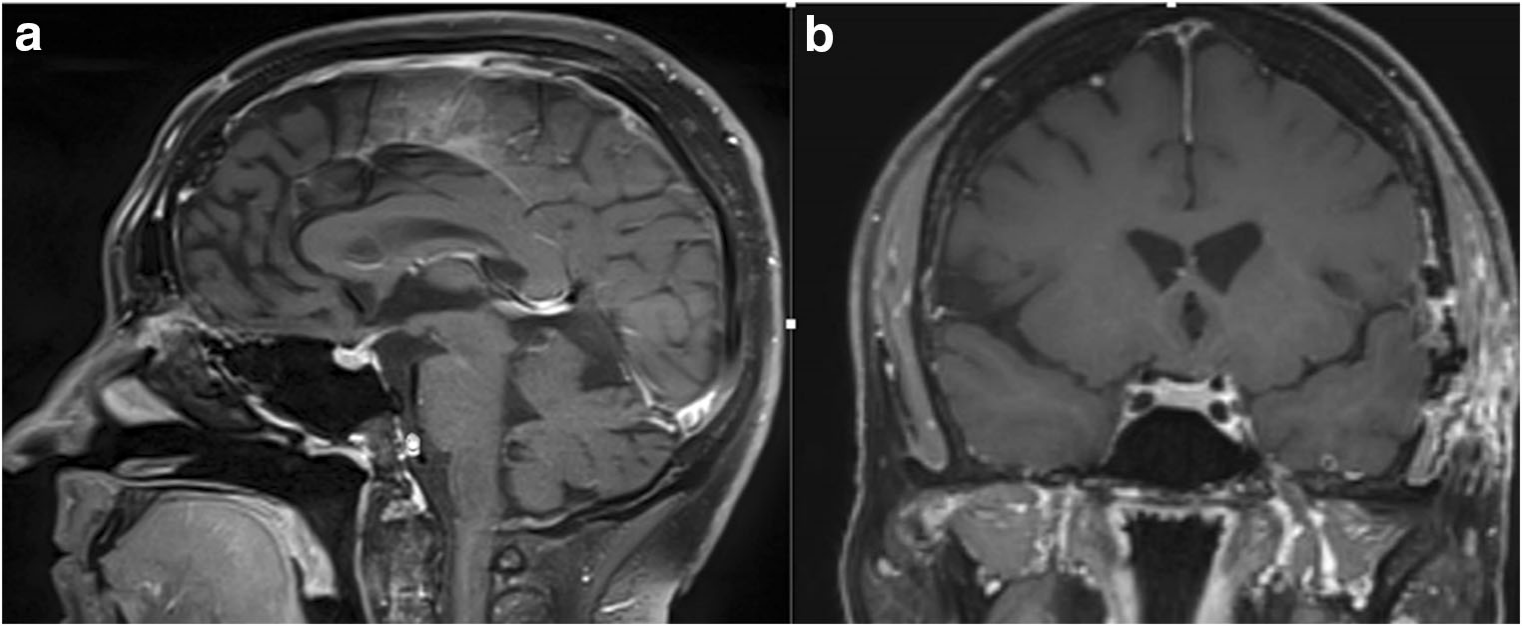
Postoperative sagittal (**a**) and coronal (**b**) MR images showing no residual tumor

The dura is opened in a “C”-shaped basal incision. The extradural clinoidectomy allows reaching the suprasellar region through a lateral subfrontal corridor with minimal brain retraction. The optico-carotid cistern, lamina terminalis, and the interoptic space are opened to let out CSF and relax the brain. The meningioma is identified and has no attachment to the planum or the tuberculum sellae. The optico-carotid and interoptic windows are developed to access the supra diaphragmatic space (Fig. 3). The meningioma is progressively debulked through these windows. The PS, SHAs, and chiasmatic perforators should be identified and protected. The dural implantation on the DS is coagulated, and the tumor capsule progressively dissected from the surrounding structures respecting the arachnoidal plane, allowing a complete resection of the DSM (Fig. 4). The relevant surgical structures such as the PS, the CNIII, ON, ICAs, SHAs, and basilar trunk are usually visualized after tumor excision. Fat graft can then be placed between coagulated dural implantation sites and the optic apparatus to allow radiosurgical treatment if necessary. A watertight dural closure is performed. The bone flap is replaced and secured using miniplates.

## Indication

DSM is a rare entity that presents with a visual disturbance in more than 90% of cases and pituitary disturbance in 60 to 90% of case [6, 7]. In symptomatic patients, surgery is considered as the first-line treatment option.

## Limitations

In case of a pure infra diaphragmatic tumor localization, the transcranial approach could be limited, and this represents an excellent indication for an endoscopic transsphenoidal approach, which allows an early and more complete resection of the dural attachment of DSM [2, 5].

Very fibrotic or calcified meningiomas, adherent to the surrounding critical structures, may represent a limitation for total resection.

## How to avoid complications

A skull base approach allows an early OC decompression and expands the surgical corridor, thereby avoiding brain and nerve retraction.

The choice of the side of approach needs to be carefully evaluated preoperatively. Unless otherwise contraindicated, the tumor is approached from the side ipsilateral to the major ON compression, in order to avoid injury of the normal ON and to provide early decompression [5].

A contralateral approach to the side of the ON compression represents as well a valid option that provides an excellent visualization of the medial aspect of the ON [5]. If the ON compression is symmetrical, a non-dominant-sided approach is performed.

Clinoidectomy and optic foraminotomy should be performed under continuous irrigation to avoid neural heat injury.

The arachnoidal plane between the tumor capsule and surrounding structure should be respected and not coagulated.

The tumor should be approached and debulked through different operative windows in order to limit excessive mobilization of the optic apparatus.

## Specific perioperative considerations

### Preoperative

Neuro-ophthalmological and endocrinological assessments are fundamental. Perioperative steroid coverage is highly recommended.

Preoperative MRI and CT scan are performed to study the bony anatomy and the pneumatization of the ACP and to evaluate the relationship of the meningioma with the PS and optic apparatus.

### Postoperative

Endocrinological assessment is fundamental. Ophthalmological follow-up is recommended at 3 months and subsequently repeated every 6 months.

Early post op MRI is needed in the first 24/72 h to evaluate the presence of any residual tumor and also to visualize the neuropexy, if performed. Follow-up MR is recommended at 3 months and then repeated annually [2, 8]. In case of a confirmed residual tumor or a recurrence, radiosurgery should be considered.

## Instructions for the post op care

A close neurological and endocrinological monitoring is necessary to evaluate the function of the optic and oculomotor nerves and to detect any signs or symptoms of pituitary dysfunction.

## Specific information to give to the patient about surgery and potential risks

Improvement of visual disturbance is expected in about 60% of cases [6, 7]. The risk of visual degradation has been reported to be as high as 34% [6, 7]. Pituitary-hypothalamic axis impairment can be seen in up 52% of patients [6, 7]. Tumor recurrence is reported in more than 15% of cases even after a GTR [6, 7]. Patients should be informed about the need for supplementary surgery or radiosurgery in case of an incomplete resection or tumor recurrence.
